# Long-term outcomes of symptomatic optic pathway glioma: 32-year experience at a single Western Australian tertiary pediatric oncology center

**DOI:** 10.3389/fonc.2023.1157909

**Published:** 2023-07-14

**Authors:** Revathi Rajagopal, Mumtaz Khan, Robert Lethbridge, Gabriel Lee, Sharon Lee, Jason Dyke, Vicki Fabian, Alycea McGrath, Mandy Taylor, Peter Jacoby, Raelene Endersby, Sumanth Nagabushan, Nicholas G. Gottardo

**Affiliations:** ^1^ Department of Global Pediatric Medicine, St Jude Children’s Research Hospital, Memphis, TN, United States; ^2^ Department of Anesthesia, Sir Charles Gairdner Hospital, Perth, WA, Australia; ^3^ School of Pediatrics and Child Health, University of Western Australia, Perth, WA, Australia; ^4^ Department of Neurosurgery, Sir Charles Gairdner Hospital, Perth, WA, Australia; ^5^ Department of Neurosurgery, Perth Children’s Hospital, Perth, WA, Australia; ^6^ Department of Neuropathology, Royal Perth Hospital, Perth, WA, Australia; ^7^ Department of Radiation Oncology, Sir Charles Gairdner Hospital, Perth, WA, Australia; ^8^ Department of Biostatistics, Telethon Kids Institute, The University of Western Australia, Perth, WA, Australia; ^9^ Brain Tumor Research Program, Telethon Kids Institute, The University of Western Australia, Perth, WA, Australia; ^10^ Kids Cancer Centre, Sydney Children’s Hospital, Sydney, NSW, Australia; ^11^ School of Women’s and Children’s Health, University of New South Wales, Sydney, NSW, Australia; ^12^ Department of Pediatric and Adolescent Oncology and Hematology, Perth Children’s Hospital, Perth, WA, Australia

**Keywords:** optic glioma, outcomes, long-term, symptomatic, visual, endocrine, second malignancies

## Abstract

**Introduction:**

Optic pathway gliomas (OPGs) are associated with significant risk of visual and endocrine morbidity, but data on long-term outcomes in symptomatic patients is sparse. This study reviews the clinical course, disease progression, survival outcomes and long-term sequelae in pediatric patients with symptomatic OPGs in our institution over three decades.

**Methods:**

Retrospective review of patients with symptomatic OPG treated in a single tertiary pediatric oncology center from 1984 to 2016.

**Results:**

A total of 37 patients were diagnosed with symptomatic OPG. Decreased visual acuity was the commonest presenting symptom (75.7%). Surgical intervention was performed in 62.2%; 56.5% underwent biopsy, 26.1% surgical debulking and 17.4% had orbital decompression with cystic fenestration and cosmetic optic nerve excision at different treatment intervals. CSF diversion was performed in 47.8% patients. Histopathologic examination confirmed 86% to be pilocytic astrocytoma and 1 ganglioglioma. 46% received chemotherapy and 48% had radiotherapy, at different intervals. Median follow-up was 13.74 years. In NF1 patients, overall survival (OS) was 100% at 5 years and 55.6 ± 24.8% at 25 years while progression-free-survival (PFS) was 50 ± 15.8% at 5 and 20 years. In non-NF1 patients, OS was 96.2 ± 3.8% at 5 years and 87.4 ± 9% at 25-years. 5-year PFS was 53.8 ± 9.8% and 25-year PFS was 49.0 ± 10%. Cumulative PFS was 53 ± 8.3% at 5 years and 49.7 ± 8.4% at 20 years while cumulative OS was 97.2 ± 2.7% at 5 years and 77.5 ± 10.8% at 25 years. 59.5% patients developed post-operative endocrinopathy. Long-term vision was normal in 8.1%, improved in 13.5%, stabilized in 40.5% but worsened in 37.8% patients. Three patients treated with radiotherapy developed second brain tumors.

**Conclusion:**

25-year OS in this cohort was 77.5% but survivorship carried significant long-term morbidities including radiation-induced second malignant brain tumors.

## 1 Introduction

Optic pathway gliomas (OPGs) represent 5% of all childhood brain tumors, with 65% presenting in children less than 5 years of age and 75% less than10 years of age. Approximately 50% are associated with neurofibromatosis type 1 (NF1) (1–3). *NF1* is a tumor suppressor gene coding for neurofibromin, a negative regulator of cell growth and proliferation *via* downstream activity of mitogen-activated protein kinase (MAPK) pathway. Mutations in this gene lead to a spectrum of sequelae, including an increased risk of malignancies (3, 4).

Although OPGs are histologically low grade, their heterogeneous clinical behavior driven by tumor proximity to vital structures, younger age at presentation and NF1 association makes a “one-size-fits-all” treatment strategy controversial (3, 4). Complete surgical resection is not considered as primary therapy due to the higher risk of surgical morbidities. Radiotherapy is reserved for salvage treatment, given its association with cognitive, endocrine, and vascular side effects even in non-NF1 patients (5, 6), making chemotherapy the favored primary treatment modality. Over the past 5 to 10 years targeted therapies using MAPK pathway inhibitors are increasingly being utilized (7–9). Chemotherapy regimens used include carboplatin or vinblastine monotherapy and carboplatin-vincristine or thioguanine-procarbazine-lomustine-vincristine (TPCV) combinations (4). Primary therapeutic indications include preservation of visual and/or hypothalamic-pituitary function (10). Despite high OS of 86-100% with multimodal treatment, OPG survivors experience significant long-term sequelae such as endocrinopathy, visual and neurocognitive deficits, vasculopathy and second malignancy (3, 5, 6). Reported incidence of late effects and long-term outcomes among OPG survivors is limited to small retrospective cohorts with short follow-up duration (11).

## 2 Methods

We performed a retrospective review of pediatric patients with symptomatic OPG diagnosed and managed at Perth Children’s Hospital (PCH) (formerly Princess Margaret Hospital for Children [PMH]), the only pediatric tertiary center in Western Australia, between January 1984 and February 2011, followed up until December 2016, to evaluate their survival rate and long-term outcomes. Patient characteristics from clinical data obtained from hospital medical records included demographics, symptom interval (time from symptom onset to diagnosis), NF1 history, clinical and visual presentations, tumor location, diagnostic detail, multimodality interventions, therapy complications and follow-up outcomes (**Tables 1**, **2**).

**Table 1:** Summary of demographics, clinical presentation, tumor location and pathology data for patients in the OPG cohort.

Patient characteristics	Non NF1 (n=26)	NF1 (n=11)	Total (n=37)
Gender			
Male	9 (34.6%)	4 (36.4%)	13 (35.1%)
Female	17 (65.4%)	7 (63.6%)	24 (64.9%)
Age at presentation			
Mean (years)	6.4	4.4	5.8
Median (years)	5.0	4.0	4.5
Range (years)	0.5-14.0	2.5-9.0	0.5-14.0
0-2 years	5 (19.2%)	0	4 (10.8%)
>2-<10years	15(57.7%)	11 (100%)	27 (73%)
>10 years	6 (23.1%)	0	6 (16.2%)
Symptoms at presentation (patient could have more than one symptom)			
Eye problems			
Decreased visual acuity	19 (73.1%)	9 (81.8%)	28 (75.7%)
Visual field defect	10 (38.5%)	0	10 (27.0%)
Nystagmus	9 (34.6%)	1 (9.1%)	10 (27.0%)
Proptosis	1 (3.8%)	4 (36.4%)	5 (13.5%)
Neurological symptoms			
Headache	8 (30.8%)	1 (9.1%)	9 (24.3%)
Vomiting	5 (19.2%)	0	5 (13.5%)
Increasing head circumference	1 (3.8%)	4 (36.4%)	5 (13.5%)
Endocrine features			
;Precocious Puberty	2 (7.7%)	1 (9.1%)	3 (8.1%)
Others			
Diencephalic syndrome	3 (11.5%)	0	3 (8.1%)
Developmental delay	2 (7.7%)	0	2 (5.4%)
Tumor location			
Optic nerve only	2 (7.7%)	5 (45.5%)	7 (18.9%)
Isolated Chiasmatic lesion	6 (23.1%)	0	6 (16.2%)
Chiasmatic and optic nerve lesions	4 (15.4%)	3 (27.3%)	7 (18.9%)
Isolated Hypothalamic lesion	1 (3.8%)	0	1 (2.7%)
Chiasmatic and hypothalamic lesions (± optic nerve)	11 (42.3%)	3 (27.3%)	14 (37.8%)
Distant metastasis	2 (7.7%)	0	2 (5.4%)
Diagnosis			
Radiological imaging only	8 (30.8%)	10 (90.9%)	18 (48.6%)
Pilocytic astrocytoma	17(65.4%)	1 (9.1%)	18 (48.6%)
Ganglioglioma	1 (3.8%)	0	1 (2.8%)

Data is shown for both non-NF1 and NF1 patients as well as for all patients (Total). The number of patients (n) is shown with percentages in parentheses.

**Table 2:** Summary of therapy administered in the OPG cohort.

Therapy at initial diagnosis	Non NF1 (n=26)	NF1 (n=11)	Total (n=37)
Observation	1 (3.8%)	10 (90.9%)	11 (29.7%)
Chemotherapy	9 (34.6%)	1 (9.1%)	10 (27.0%)
Radiotherapy	11 (42.3%)	0	11 (29.7%)
Surgery	5 (19.2%)	0	5 (13.5%)
Overall treatment			
Observation only	0	6 (54.5%)	6 (16.2%)
Chemotherapy only	6 (26.9%)	0	6 (16.2%)
Radiotherapy ± chemotherapy	14 (53.8%)	4 (36.4%)	18 (48.6%)
Surgery ± chemotherapy	5 (19.2%)	0	5 (13.5%)
Surgery, radiotherapy ± chemotherapy	1 (3.8%)	1 (3.8%)	2 (5.4%)
Chemotherapy			
1^st^ line of chemotherapy	15	2	17
2^nd^ line of chemotherapy	5	1	6
3^rd^ line of chemotherapy	1	1	2
4^th^ line of chemotherapy	0	1	1
5^th^ line of chemotherapy	0	1	1
Diagnostic and therapeutic surgical procedure for OPG (patient could have more than one procedure)	21 (80.8%)	2 (18.2%)	23 (62.2%)
Biopsy	12	1	13
VP shunt/ventriculostomy	10	1	11
Debulking	6	0	6
Cystic fenestration	2	0	2
Optic nerve decompression	1	1	2

Data is shown for both non-NF1 and NF1 patients as well as for all patients (Total). The number of patients (n) is shown with percentages in parentheses.

Progression-free survival (PFS) and overall survival (OS) were analyzed to evaluate survival outcomes. OS was defined as the time interval from diagnosis to death from any cause or the interval from diagnosis to last follow-up for patients who are alive. PFS was defined as the time interval from treatment to the time of disease progression or recurrence, to the last follow-up, or to death from any cause.

Neuroimaging, ophthalmologic and neuroendocrine data were reviewed to assess long-term outcomes. Visual and neuro-endocrine assessments were performed at diagnosis, during and after treatment. Teller Acuity Cards and Snellen charts were used to assess visual acuity (VA) in preverbal and verbal patients respectively. Visual fields (VF) using visual perimetry was recorded if available. VA was the primary visual outcome and VF considered as additional information, based on the Response Evaluation in Neurofibromatosis and Schwannomatosis International Collaborative Group in OPG (REiNS) criteria (10, 12). VA data was categorized according to the International Council of Ophthalmology guideline as normal vision (≥20/25), mild impairment (20/32-20/63), moderate impairment (20/80-20/160), severe impairment (20/200-20/400), profound impairment (20/500-20/1000), near blindness (<20/1000) and blindness (no perception of light [NPL]) (13). For the purpose of this analysis, visual outcome was grouped into normal vision, normal visual acuity with VF defect, mild to moderate visual acuity impairment (20/32-20/160) with or without VF defect and legally blind at least in one eye (≤20/200). Radiotherapy medical records were used to analyze radiation characteristics and outcomes.

### 2.1 Statistical analyses

Data was collected and presented as total number, percentage, mean, and median, conforming to data type. Survival analysis was performed using the Kaplan-Meier method. The log-rank test was used to detect significant difference between 2 or more survival functions and p values < 0.05 were regarded as statistically significant. Chi squired test was used to investigate associations between variables. Analyses were performed using SPSS version 20.

## 3 Results

A total of 37 patients presenting with symptomatic OPG were identified, of which 11 (29.7%) had NF1. Female: male ratio was 1.8:1 overall (24 female, 13 male) as well as for NF1 patients (7 female and 4 male). Combined median age at diagnosis was 4.5 years (range 0.5 – 14 years). Non-NF1 patients were distributed across all three age categories, median age 5 years (range 0.5 – 14 years); while NF1 patients were 2.5 to 9 years old, median age 4 years (range 2.5 – 9 years) (**Table 1**).

Symptom interval was variable across the cohort. In 6/37 (16%) of patients it was more than 52 weeks, 4 to 52 weeks in 21/37 (56.8%), 1 to 4 weeks in 6/37 (16%), and more than 7 days in 4/37(10.8%) of patients. The most common presenting symptom was decreased visual acuity, found in 28/37 (75.7%) of patients. Precocious puberty was the only presenting endocrinopathy, extant in 3/37 (8.1%) of patients. Non-NF1 patients presented more commonly with headache and nystagmus, while NF1 patients presented with proptosis and increasing head circumference (**Table 1**).

Magnetic resonance imaging (MRI) and computed tomography (CT) was performed at presentation for 22/37 (59.5%) patients, 9/37 (24.3%) had MRI alone and 6/37 (16%) had CT scan leading to diagnosis. All six patients who had CT underwent subsequent MRI before treatment. Isolated optic nerve involvement was present in 7/37 (18.9%), while 6/37 (16.2%) had only chiasmatic involvement, 7/37 (18.9%) demonstrated optic chiasmatic and nerve lesions, 14/37 (37.8%) had chiasmatic and hypothalamic involvement and 3% (1/37) had isolated hypothalamic involvement. Two patients (5.4%) presented with spinal metastasis, diagnosed following spinal MRI (**Table 1**). Upfront surgical intervention was performed in 19/37 (51.4%), while 18/37 (48.6%) of patients were diagnosed with an OPG based on imaging features alone. Histological diagnoses underwent retrospective central review by two independent pathologists. All but one of the biopsied tumors were pilocytic astrocytomas (86%) while the other was a ganglioglioma.

Management was varied and influenced by the presence of NF1. Visual deterioration was the most common indication for therapy. Of the 11 NF1 patients, 91% underwent observation alone while only one received chemotherapy at diagnosis, second-line chemotherapy for recurrence and eventually radiotherapy eight years from diagnosis. Of the observed NF1 patients, 40% progressed and received treatment with radiotherapy, chemotherapy and/or surgery. The mean time from diagnosis to treatment was 1.1 year (range 1.0 - 2.5 years). Of the 26 sporadic (non-NF1) patients, all but one (96.1%) received treatment at diagnosis within a median time of 1 month (range 0 - 3.5 months): 42.3% were irradiated, 34.6% received chemotherapy and 19.2% had surgery (**Table 2**). The non-NF1 patient who was initially observed only, developed progressive disease 42 months after diagnosis and subsequently received radiotherapy.

Vincristine and carboplatin combination was the most common first-line chemotherapy regimen, used in 17/37 (45.9%) patients either at diagnosis or following progression after surgical intervention or radiotherapy. A total of 20/37 (54%) received radiation at different time-points; 11/20 (55%) at diagnosis, 6/20 (33%) at first progression, 2/20 (10%) at second progression and one (5%) had radiotherapy during 4^th^ disease progression. The median age for radiation was 7.1 years (range 2.5-14.2). A median dose of 54 Gy (range 50 - 72 Gy) delivered in daily fractions of 1.8 Gy (range 1 - 2 Gy).

Overall, surgical interventions were performed in 23/37 (62.2%) at different intervals for diagnostic, symptomatic relief, and therapeutic indications. Of these 13/23 (56.5%) underwent diagnostic biopsy, 6/23 (26.1%) debulking surgery and 2/23 (8.7%) had cosmetic orbital decompression for severe proptosis. Primary cerebrospinal fluid diversion by ventricular peritoneal shunt or third ventriculostomy was performed in 11/23 patients (47.8%). Two (8.7%) patients had cystic fenestration during follow-up for visual pathway decompression and intracranial pressure relief.

### 3.1 Survival outcomes

**Figure 1:**
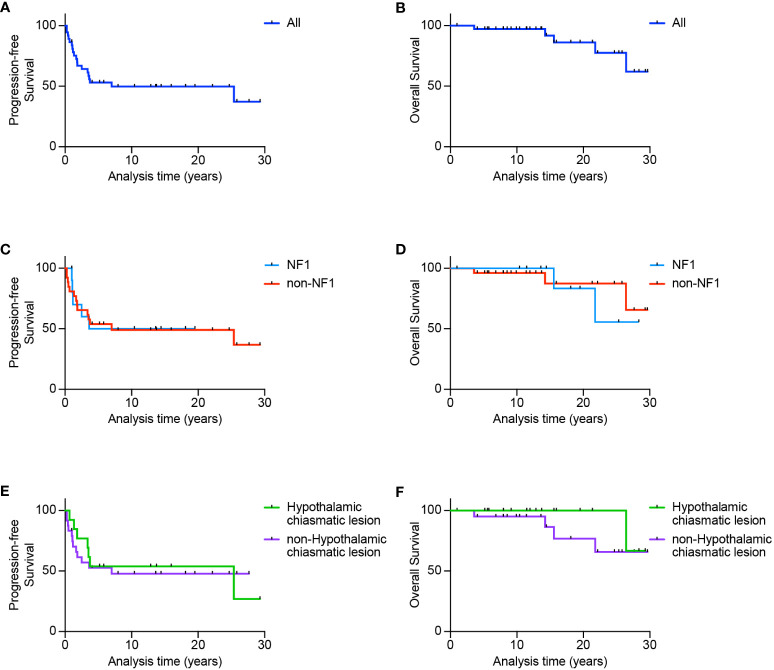
Survival outcomes for patients diagnosed with optic pathway glioma. Progression-free survival **(A)** and overall survival **(B)** for the whole cohort. Also shown is progression-free and overall survival according to NF1 status **(C, D)** or according to either hypothalamic-chiasmatic vs non-hypothalamic-chiasmatic location **(E, F)**.

Eleven patients were diagnosed before 1991, six between 1991 and 2000 and 20 between 2001 and 2011. Median follow-up for all patients was 13.74 years (range 1.0 - 29.64). PFS for the whole cohort was 53 ± 8.3% at 5 years and 49.7 ± 8.4% at 10, 20 and 25-years (**Figure 1A**) while OS was 97.2 ± 2.7% at 5 and 10 years, 86.1 ± 7.8% at 20 years and 77.5 ± 10.8% at 25 years (**Figure 1B**). PFS for the NF1 group was 50 ± 15.8% at 5, 10 and 20 years while the non-NF1 group demonstrated a 5-year PFS of 53.8 ± 9.8% and 49.0 ± 10% at 10, 20 and 25-years (**Figure 1C**). The 5 and 10-year OS in NF1 patients was 100%, 20-year was 83.3 ± 1.5% and 25-year was 55.6 ± 24.8%, whilst for non-NF1 patients, 5 and 10-year OS was 96.2 ± 3.8% and 87.4 ± 9% at 20 and 25-years (**Figure 1D**). There were no statistically significant differences in PFS (p = 0.91) or OS according to NF1 status (p = 0.65).

Of the 19 (51.4%) patients who demonstrated progressive disease, 84.2% had local progression at recurrence, 5% developed local and leptomeningeal progression, and 10% with spinal metastasis at diagnosis developed both local and disseminated disease. 17/19 (89%) of patients progressed <4 years from initial diagnosis with a median of 1.6 years (range: 0.3-25.4). All but 4/5 (80%) patients ≤2 years of age developed disease progression, compared with 15/32 (46.9%) > 2 years of age, however, this just failed to reach statistical significance (p=0.053). A total of 18/37 (48.6%) of patients remain alive without progression. Five (13.5%) patients died, including two with NF1. Causes of death included sepsis (n=2), second malignant brain tumors (n=2), and progressive disease (n=1).

Based on location, tumors were classified *hypothalamic-chiasmatic* if both structures were involved, with no obvious distinction. In contrast, tumors were classified *non-hypothalamic-chiasmatic* if they were limited to the optic chiasm or nerve, or exhibited distant metastasis excluding the hypothalamic or chiasmatic regions. For hypothalamic-chiasmatic tumors, PFS plateaued at 50 ± 12.5% 4 years post-diagnosis until 25 years, while 5-year PFS was 55.7 ± 11.1% and 10, 20 and 25-year PFS was 50.1 ± 11.3% (**Figure 1E**) for non-hypothalamic-chiasmatic tumors, although this difference was not statistically significant (p = 0.97). For hypothalamic-chiasmatic tumors the 5, 10, 20 and 25-year OS was 100%. While for the non-hypothalamic group, the 5, 10, 20 and 25-year OS was 95 ± 5%, 86.4 ± 9%, 76.8 ± 12.3% and 65.8 ± 14% (**Figure 1F**).

### 3.2 Endocrinological outcomes

**Figure 2:**
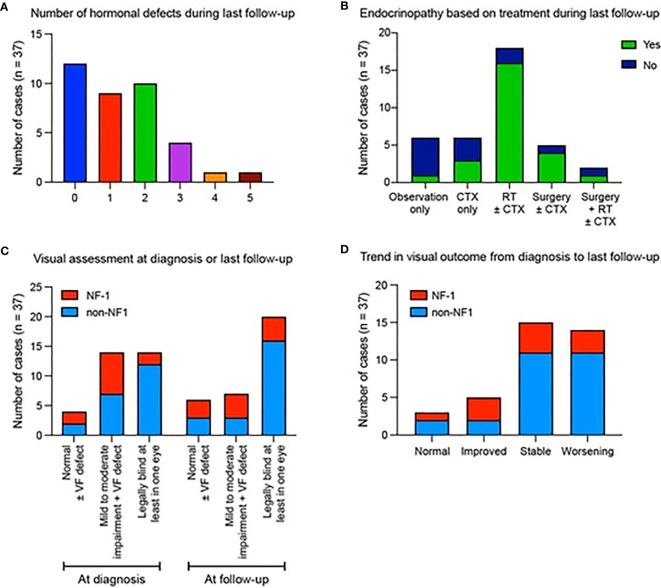
Summary of hormonal defects and visual assessments for patients diagnosed with optic pathway glioma. Data show **(A)** the number of hormonal defects observed in patients at their most recent follow-up. **(B)** The presence (green) or absence (blue) of endocrinopathies depending on the type of therapy received. **(C)** Visual outcome according to NF1 status at diagnosis and final follow up. **(D)** Trend in visual outcome according to NF1 status from diagnosis to last follow-up.

Of the 37 patients, 3 patients (8.1%) presented with precocious puberty at diagnosis which required upfront hormonal therapy. Endocrinopathy developed in 22/37 (59.5%) patients following therapy without prior evidence of hormonal defects at diagnosis. Overall, 9/37 (24%) had one hormonal insufficiency, 10/37 (27%) had two, 4/37 (10.8%) developed three defects and 2/37 (5.4%) displayed panhypopituitarism (≥4 anterior pituitary hormonal defects) (**Figure 2A**). Precocious puberty was the most common endocrinopathy, seen in 13/37 (35.1%), followed by growth hormone insufficiency in 12/37 (32.4%), hypogonadism in 11/37 (29.7%), hypothyroidism in 11/37 (29.7%), low cortisol in 4/37 (10.8%), and diabetes insipidus in 2/37 (5.4%). No endocrine complications were seen in five patients who underwent observation alone (16.2%). Endocrinopathy was significantly more common in 17/20 (85%) patients who received radiotherapy versus 6/17 (35.3%) those who did not receive radiotherapy (p = 0.003) (**Figure 2B**), and in 14/16 (87.5%) of patients with hypothalamic-chiasmatic lesions versus 11/21 (52.3%) in those without hypothalamic-chiasmatic lesions (p=0.024). Surgery and NF1 status were not associated with endocrine dysfunction.

### 3.3 Visual outcomes

Visual symptoms were the commonest clinical presentation, with 14/37 (37.8%) of patients legally blind in at least one eye at presentation, 14/37 (37.8%) with mild-moderate VA impairment and 5 (13.5%) having VF defect with intact VA (**Figure 2C**), with these proportions being 20/37 (54.1%); 7 (18.9%) and 4 (10.8%) at final follow-up, respectively. At diagnosis 9/16 (56.2%) of patients with hypothalamic-chiasmatic tumors and 5/21 (23.8%) with non-hypothalamic-chiasmatic lesions were legally blind in at least one eye. By final follow-up, an additional two (68.8%) patients with hypothalamic-chiasmatic and four (42.9%) with non-hypothalamic-chiasmatic patients became legally blind in at least one eye. At last follow-up, normal vision was observed in 3/37 (8.1%); improved in 5/37(13.5%); remained stable in 15/37 (40.5%) and worsened in 14/37 (37.8%) (**Figure 2D**). No statistically significant association was identified between visual outcomes and treatment, age, NF1 status or tumor location, likely due to relatively low sample numbers.

### 3.4 Secondary brain tumors

**Figure 3:**
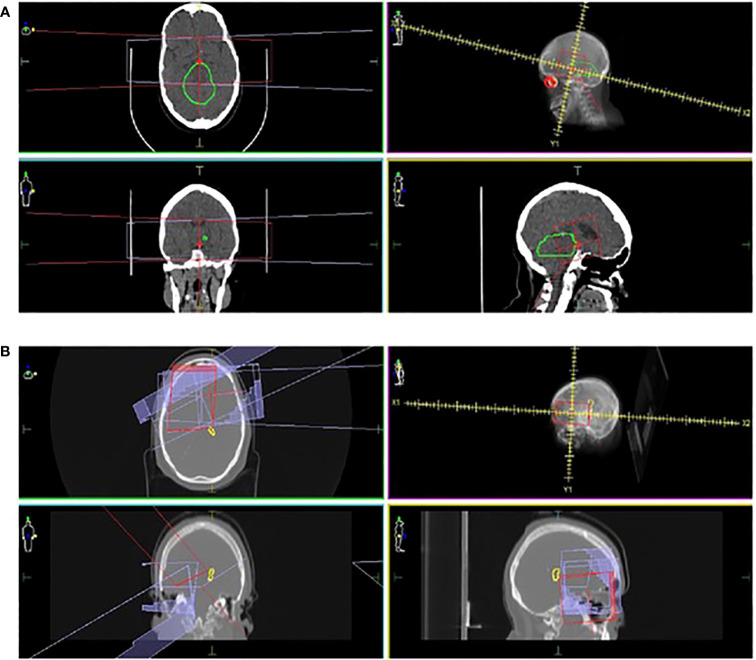
Locations of second brain malignancies relative to the treatment field. CT images in three planes, radiation treatment plans and the locations of second brain tumors are shown. In one patient **(A)**, the second tumor (marked as green) is observed within the radiation field (marked in red and purple). In a second case **(B)**, an anaplastic astrocytoma (marked as yellow) occurred on the edge of the radiation field (marked in red and purple).

Three patients (8.1%) developed a secondary brain tumor, presenting at 20.6, 25.3 and 28.7 years after primary diagnosis, with a 30-year cumulative incidence of 9.1. All three patients had received radiotherapy, with a 25-year median interval between radiotherapy and second brain tumor diagnosis. In one patient, the second malignancy occurred within the original radiotherapy field (50Gy; 30 fractions) in the brainstem (**Figure 3A**). The patient received stereotactic radiotherapy for a presumed high-grade glioma but died following aspiration pneumonia. One patient had NF1 and the patient with NF1 was treated with 72 Gy (60 fractions) and subsequently developed a histologically proven anaplastic astrocytoma on the edge of the radiotherapy field (**Figure 3B**). The patient received re-irradiation with concurrent temozolomide but died of progressive disease one year later. The third patient developed a meningioma but further outcomes, including radiation field involvement, are unknown.

### 3.5 Vascular outcomes

Two patients (18%) with NF1 developed vasculopathies. One patient who was observed only, acquired Moya Moya syndrome one-year post-diagnosis. Another patient developed vasculopathy 7 years post-radiotherapy.

## 4 Discussion

The recent dramatic increase in knowledge of the molecular biology of OPGs has impacted diagnosis and management, resulting in 90% OS but patients still carry significant risk of long-term visual, hormonal, and neuro-cognitive sequelae (14). There is particular paucity of studies examining symptomatic OPGs. This single center retrospective study addresses this gap by investigating survival and morbidity in 37 Western Australian children with symptomatic OPGs over a 32-year period, with a minimum of 5 years follow up. To our knowledge, this review represents the longest reported follow-up to date for children with OPG.

Comparable with previous reports, 29.7% had NF1 (1, 2, 6). Our patient cohort consisted of 64.1% females, although OPGs do not appear to show clear gender predilection (1, 3, 6, 15). The median age of diagnosis for the whole cohort was 4.5 years; with a slightly younger median age of 4 years for NF1 patients compared with their sporadic counterparts (5 years). The reasons for a lower median age of diagnosis in NF1 patients may be due to NF1 screening programs (14). Notably, all children with NF1 associated OPG’s presented with age >2 years while 19% of non-NF1 associated OPG’s were observed in children <2 years old, who also demonstrated higher incidence of progression, consistent with other reports (3, 7, 16–19), possibly driven by yet unclear molecular mechanisms.

Sporadic OPGs were seen more in hypothalamic-chiasmatic locations (42.3%), whilst optic nerve involvement was more common in NF1 patients, aligning with previous reports (14). No significant OS and PFS differences were observed between the two location-based groups and these findings were in contrast to the report from Fouladi M et al. (20). The majority of our patient cohort presented with visual symptoms, including defective acuity (VA) and fields (VF), nystagmus, and headaches. 51.4% of patients had histological diagnoses while 48.6%% were radiologically confirmed. As expected, pilocytic astrocytoma was the commonest histological subtype (5, 20).

Management varied throughout the 32-year study period. Surgical procedures included biopsy, CSF diversion, cyst drainage and partial resection intending optic pathway decompression and vision preservation. Most of the patients undergoing CSF diversion underwent opportunistic biopsies. Biopsy was performed if there was radio-diagnostic dilemma. One patient underwent a cosmetic orbital decompression, and the lesion was histologically proven to be a ganglioglioma. There is no established surgical standard of care for OPGs, due to potentially significant neurological morbidity to the visual pathway, vital brain structures and fatality, mandating a highly individualized approach (21).

Although radiotherapy can be a useful adjuvant in OPG management, its association with endocrinopathy, neurocognitive dysfunction, cerebral vasculopathy and second primary brain tumors, particularly in NF1 patients, limits its role in frontline therapies (5, 6, 22, 23) as well as subsequent therapy for progression, especially in children who have not completed growth and pubertal development. In our study, radiotherapy was more frequently employed in the first half of study period (1980 and 1990’s), but the practice shifted towards radiotherapy sparing approach during the last decade to minimize radiotherapy related morbidities. Multiple studies have shown that chemotherapy is the treatment of choice, especially in young children with OPG (1, 4, 6, 10). Radiotherapy is preferred in older patients with chemotherapy-refractory disease and those less susceptible to radiotherapy toxicity (5). However, several retrospective studies have reported that upfront chemotherapy was associated with inferior PFS, especially in children younger than < 6 years old compared to older patients (5, 20). This finding suggests that infants and toddlers may have more aggressive tumor biology and/or resistance to conventional chemotherapy agents which resulting in inferior outcomes (5). Furthermore, other studies have shown that VA was worse among children with OPG who received first-line chemotherapy compared to those who received radiotherapy as the primary treatment (24, 25). Hence, the decision to commence upfront radiotherapy must be balanced by weighing risks and benefits between disease related morbidity and treatment related toxicity in a multidisciplinary team setting.

Several chemotherapeutic agents were used in different regimens. These included carboplatin and vincristine, single agent vinblastine, TPCV, etoposide, ifosfamide, and bevacizumab containing regimens. A total of 46% received first-line chemotherapy and 16.2% received second-line chemotherapy due to progression. Third-line chemotherapy was required in 5.4%, while 2.7% needed fourth-line chemotherapy. Comparative effectiveness between regimens is difficult due to variation in age of onset, location, and extension of tumor, newly diagnosed versus recurrent tumor, NF1 status and radiological definition of disease response and progression. Overall, chemotherapy stabilizes disease progression and visual impairment but confers short and potentially long-term toxicity (10, 26).

Previous studies have reported similar PFS at 5, 10, 20 and 25-year time points to our cohort (14, 22, 27). However, linear comparison across those studies was limited by differences in age groups (5, 6, 20). In our study, 89% of patients who had disease progression demonstrated first progression within four years from diagnosis. PFS didn’t differ between hypothalamic-chiasmatic and non-hypothalamic-chiasmatic locations and NF1-OPGs tended to have a less aggressive course, with a lower rate of progression, as previously reported (28, 29). While this was not found to be statistically significant within our cohort, it is consistent with other studies [1,6]. OS of NF1-OPGs at 5, 10, 20 and 25 years correlates with other studies (14, 20, 27, 30). Only one death was directly related to progressive disease (1/5; 20%), with the majority due to treatment-associated complications (4/5; 80%), perturbingly 50% (2/4) of these where due to second malignant neoplasms approximately two decades following treatment with radiotherapy.

Previous small retrospective cohorts, with follow-up periods <10 years, have reported endocrinopathies ranging from 27 to 100% (3, 11). In our study, a significant proportion (59.5%) without initial evidence of endocrinopathy developed post-therapy hormonal defects, especially among those who received radiotherapy (85%). Hypothalamic-chiasmatic location was a predictor for endocrinopathy, the first endocrine event occurring at a median 0.8 (0 – 14.2) years from diagnosis, as reported before (8). Precocious puberty was the most common hormonal defect in our cohort (64.8%), although growth hormone deficiency has been frequently reported (11, 20, 31). Etiology is unclear but tumor location, growth, and radiotherapy <5 years old are likely contributors (32).

Visual preservation is the mainstay of OPG management; however, visual deterioration remains a major risk. In our cohort, 8.1% of patients maintained normal vision, 13.5% had visual improvement, 40.5% had stable vision and 37.8% had worsening vision. This was consistent with several studies which have reported visual decline in 37.8 to 41% of patients despite conventional therapy (10, 19, 33, 34). The relatively low patient numbers combined with the heterogenous nature of our cohort, precluded our ability to discern the impact of radiotherapy versus chemotherapy on visual outcomes. In addition, inconsistencies in inter-study outcomes relate to varied patient characteristics, tumor location, treatment modalities and difficulties in visual assessment for children < 5 years. Studies have shown that serial VA changes do not reliably identify tumor progression and tumor progression does not correlate well with decreased VA, while better initial VA, older age, absence of post-chiasm tumor and presence of NF1 were associated with improved or stable VA outcomes (19, 35, 36).

In our cohort, three patients who received radiation developed a second brain tumor more than 20 years post-diagnosis, with two being in-field occurrences. The 30-year cumulative incidence was 9.1%, while others have reported a very similar incidence over 10 years (5). Previous studies describe a 6–9-year median latent interval between radiotherapy and onset of a second CNS malignancy (5, 37). In our cohort, the median latent interval was much longer (25 years), highlighting the ongoing risks of radiotherapy as a treatment modality for these tumors. Data relating to radiotherapy associated toxicities are largely from historical experiences between 1970 and 1990’s (38). Recent advancements in modern radiotherapy techniques, especially intensity modulated radiotherapy (IMRT) and proton therapy, have enabled safer and effective treatment by sparing normal organs (38, 39). In the Children’s Oncology Group, ACNS0221 study, 71% of patients received IMRT and the result showed favorable disease control and low toxicity without marginal failures (40). In terms of proton therapy, Indelicato et al. conducted a prospective study in pediatric low-grade glioma (pLGG) and confirmed that proton therapy was an effective treatment in pLGG without local failures while toxicity profiles were tolerable (41). Reduction in total integral dose delivery to normal tissue without compromising the target volume coverage significantly reduced the risk of secondary cancer with proton therapy (42). However, clinical data are limited, and long-term follow-up is recommended to monitor the incidence of proton therapy related late effects (41, 42).

The development of vasculopathy is highly dependent on tumor location especially in OPG due to anatomic proximity to the circle of Willis (5). Furthermore, vascular abnormality is a well-recognized manifestation in patients with NF1 and radiotherapy is associated with an increased risk in the incidence of serious vasculopathy in OPG patients with underlying NF1. The 10-year cumulative incidence of vasculopathy among children with OPG was reported as 7.1% and in this study, vasculopathy did not develop in children aged >10 years old at the time of receiving radiotherapy (5). Merchant et al. also found a higher incidence of vasculopathy in pLGG when radiotherapy was delivered to children <5 years old compared with children >5 years old (12.5% vs. 3.8%) (43). Thus, younger patients with OPG are at higher risk of vasculopathy regardless of modern radiotherapy techniques (proton or photon) due to exposure of radiotherapy target prescription dose to large intracranial arteries especially circle of Willis in relation to tumor geometry (5, 39).

Our data reinforces previous reports and highlights the urgent need to consider novel molecularly selective strategies. Knowledge of the molecular genomics of pLGG has grown exponentially over the last 2 decades, with evolution of new treatment options.

Somatic activating mutations in the mitogen activated protein kinase (MAPK) cellular signaling pathway play a central role in upregulation of downstream transcription effectors *via* receptor tyrosine kinases (44, 45). The BRAF proto-oncogene is the most frequently altered in pLGGs, with BRAF-KIAA1549 fusion and BRAFV600E the most common molecular alterations (44, 46). These biological insights have led to the introduction of novel molecular-selective therapies, targeting genes such as BRAF (e.g., vemurafenib and dabrafenib), MEK (e.g., trametinib and selumetinib) and FGFR (e.g., erdafitinib and infigratinib), or agents targeting signaling pathways like pan-RAF inhibitors (tovorafenib (DAY101)) and mTOR pathway inhibitors (everolimus) (7–9, 47–49). Ongoing and upcoming trials (e.g., NCT02285439) are investigating the efficacy of some of these targeted therapies as monotherapy (e.g., NCT04775485) as well as in combination with conventional chemotherapeutic agents. (e.g., NCT03871257, NCT04166409, NCT04576117). However, care must be exercised to ensure paradoxical activation does not occur (50). MAPK activation can also occur due to underlying genetic predisposition to OPG as is the case with NF1, which has its own distinct biology, treatment options and responses compared to sporadic OPG’s (51).

We acknowledge that our study is limited by its single-institutional retrospective nature, relatively small sample size and considerable variation in management approaches over three decades, restricting direct comparisons with published evidence. Neurocognitive outcomes and molecular pathology data (eg, BRAF mutations and fusions) were not available and hence not included in the analyses.

## 5 Conclusion

Pediatric OPGs are challenging tumors to manage, with limited reports for long-term outcomes in symptomatic lesions. Our study provides a valuable addition to bridging this gap, describing demographics, clinical presentation, treatment, and long-term outcomes over a period of 32 years. Younger age patients (<2 years old) are at risk for an inferior outcome. Radiotherapy increased the risk of developing endocrinopathy and second primary brain tumors. Notably, second primary brain tumors were the cause of 40% of deaths for the whole cohort. Despite multimodal conventional therapies, visual impairment was a significant contributor to long-term morbidity, with almost 40% of patients sustaining visual decline. This study highlights the need for meticulous long-term surveillance and advocates for further refinement of therapeutic options aided by a precision oncology approach, through prospective clinical trials.

## Data availability statement

The original contributions presented in the study are included in the article/Supplementary Material. Further inquiries can be directed to the corresponding author.

## Ethics statement

This retrospective audit was approved by the Child and Adolescent Health Service, Quality and Safety Committee (Quality Activity number 1776) with delegated authority from the Princess Margaret Hospital Human Research Ethics Committee, within which the work was undertaken. It conforms to the provisions of the Declaration of Helsinki in 1995 (as revised in Tokyo 2004) and the National Statement on Ethical Conduct in Human Research. Direct consent was obtained from the parents of some children to access clinical information in those cases where these patients were followed up by clinicians outside our institution.

## Author contributions

MK and RL contributed equally. Drafting of the paper: MK, RL, RR and SN. Care of patients and provision of clinical data: NG, GL, SL, JD, VF, AM, MT, PJ. Data analysis and graphics generation: RR, RE Senior authorship: SN, NG. All authors reviewed and edited the paper. All authors contributed to the article and approved the submitted version.
